# Safety of Early Weight Bearing Following Fixation of Bimalleolar Ankle Fractures

**DOI:** 10.7759/cureus.7557

**Published:** 2020-04-06

**Authors:** Braden J Passias, Frederick P Korpi, Anson K Chu, Devon M Myers, Greg Grenier, David K Galos, Benjamin Taylor

**Affiliations:** 1 Orthopedic Surgery, OhioHealth, Columbus, USA; 2 Orthopedic Trauma, Spectrum Orthopedics, Canton, USA; 3 Foot and Ankle Surgery, OhioHealth, Columbus, USA; 4 Orthopedic Trauma, Nassau University Medical Center, Great Neck, USA; 5 Orthopedic Trauma, OhioHealth Grant Medical Center, Columbus, USA

**Keywords:** ankle fracture, bimalleolar ankle fracture, weight bearing, medial clear space

## Abstract

Ankle fractures are common orthopedic injuries. Although operative indications and subsequent stabilization of these fractures have not significantly changed, postoperative protocols remain highly variable. Effects of early weight bearing (EWB) on fracture characteristics in operatively stabilized bimalleolar and bimalleolar equivalent ankle fractures remain poorly publicized. This study seeks to clarify postoperative fracture union rates, rates of hardware loosening or failure, and radiographic medial clear space changes when comparing EWB to late weight bearing (LWB) following open reduction and internal fixation (ORIF).

A total of 95 patients with either bimalleolar (66%) or bimalleolar equivalent (34%) fractures who underwent ORIF were retrospectively reviewed. Weight bearing was allowed at three weeks in the EWB group and when signs of radiographic union were noted in the LWB group. Postoperatively, patients were evaluated at regular intervals for fracture union, signs of implant failure, and evidence of medial clear space widening radiographically.

There were 38 patients (40%) in the EWB group and 57 patients (60%) comprising the LWB cohort. There were no significant demographic differences between groups. The EWB group on average began to weight bear at 3.1 + 1.4 weeks postoperatively, whereas the LWB group began at 7.2 + 2.1 weeks postoperatively (p<0.01). Union rate (p=0.51), time to union (p=0.23), and implant failure (p>0.1 at all time intervals) were not notably different between groups. No differences in medial clear space were detected at any postoperative interval between groups (p>0.1 at all time intervals). This study suggests that EWB at three weeks postoperatively does not increase markers of radiographic failure compared to six weeks of non-weight bearing (NWB), which has been regarded as the gold standard of treatment to allow for healing; this may represent an improvement to rehabilitation protocols after bimalleolar ankle ORIF of unstable ankle fractures.

## Introduction

The incidence of ankle fractures is approximately 71-187 per 100,000 people per year and is one of the most common injuries treated by orthopedic surgeons [1-5]. The incidence is likely to increase as the average age of the population rises, as does the amount of participation in sports-related activities [6,7]. The majority of ankle fractures occur secondary to ground-level falls, but irrespective of the injury mechanism, fracture characteristics often dictate the need for operative stabilization [3]. Although operative guidelines are fairly well established, postoperative weight-bearing protocols have not been well studied, and controversy exists regarding optimal time to weight bearing in this population.

Osteosynthesis techniques for bimalleolar ankle fractures are relatively well established, but operative planning can be dependent on fracture pattern, surgeon preference, and patient comorbidities. Various fixation strategies for lateral malleolar fractures have been described; lag screws with neutralization plating, intramedullary devices, antiglide plating, and bridge plating in cases of comminution are some of the most commonly used techniques in practice today. Similarly, screws, tension band constructs, or buttress plating techniques can be used for stabilization of medial malleolus fractures [8,9]. Traditionally, after ankle open reduction and internal fixation (ORIF), six weeks of non-weight bearing (NWB) was thought to be the gold standard to allow optimal immobilization for healing [10,11]. The theoretical risk of fixation failure and loss of reduction secondary to inadequate immobilization and early weight bearing (EWB) drove this traditional protocol [12].

Recent research has shown that extended periods of immobilization can lead to complications such as joint stiffness, ligamentous and musculotendinous atrophy, increased time for return to work, and difficulties with activities of daily living (ADLs) [9,11,13]. Difficulties with ADLs specifically include inability to drive, limited mobility, and difficulties with hygiene, which all presumably have deleterious effects on patient outcomes. More recently, however, early studies have begun to look at the benefits of early mobilization among ankle fractures after ORIF. Analysis of this contemporary ideology has yielded multiple biomechanical and cellular advantages. EWB may also provide a more functional ankle joint and an earlier return to work [9,11,13].

There has been a trend in the literature towards attempting to clarify outcomes of EWB in lower extremity fractures treated operatively. Immediate, EWB and LWB protocols have all been reviewed, as have different methods of immobilization in such groups [10,14-17]. Although these studies are helpful in comparing various postoperative courses, there still exists a paucity in the literature that adequately describes the safety and efficacy regarding EWB of ankle fractures, specifically bimalleolar variants [18]. The limited data available on time to weight bearing in bimalleolar fractures have revealed conflicting results, with some showing a correlation between EWB and improved functional outcomes, and others demonstrating no difference when juxtaposing long-term effects [19,20]. 

The purpose of this study is to better understand the advantages and disadvantages of EWB following ORIF of bimalleolar ankle fractures. This study provides a review of outcomes associated with a more aggressive postoperative protocol following bimalleolar ankle fracture fixation where patients are allowed to weight bear at three weeks postoperatively [4,8,11,19-25]. We hypothesized that EWB following bimalleolar ankle fracture fixation would not negatively affect postoperative radiographic alignment, union rates, or rates of complication which included nonunion, malunion, and hardware failure.

## Materials and methods

Following formal Institutional Review Board approval, a retrospective review was conducted on all patients with operatively managed bimalleolar ankle fractures between 2010 and 2015. Each patient was treated by one of two fellowship-trained orthopedic trauma surgeons at an urban level 1 trauma center. Based on training and clinical experience, the two surgeons participating in this study differ in their postoperative weight-bearing protocols following ORIF of ankle fractures; one surgeon allows EWB at three weeks postoperatively, while the second continues strict NWB for at least six weeks postoperatively.

Inclusion criteria for ORIF included bimalleolar ankle fractures and those so-called bimalleolar equivalent fractures, with lateral malleolus fractures and associated medial clear space widening on radiographic stress examination due to medial soft tissue injury. Furthermore, only patients with closed fractures and a complete imaging series, including manual stress testing for isolated lateral malleolar fractures, were included. At least one year of follow-up was mandatory for inclusion. Patients with open fractures, polytrauma injuries, posterior malleolar fractures, open deltoid ligament repair, or syndesmotic fixation were excluded from the study. The exclusion of open fractures allowed investigators to assess low-energy ankle injuries with a minimal amount of soft tissue attenuation, which could have had a varying effect on healing rates regardless of postoperative weight-bearing protocol. Also excluded were patients with medical comorbidities such as diabetes or peripheral neuropathy that would preclude them from immediate weight bearing.

During surgery, participants underwent rigid operative fixation of bony injuries including the lateral malleolus and medal malleolus if present. The lateral malleolus was fixed using either a lag screw and neutralization plate, antiglide plating, or a bridge plate technique when comminution was present; the medial malleolus fragment was secured by two 3.5 or 4.0 mm screws of appropriate length or a medial minifragment plate in cases of comminution. In cases of bimalleolar equivalent fractures, the lateral malleolus was fixed in stable position and the ankle was stressed under fluoroscopy. Radiographs were evaluated for evidence of residual talar tilt, medial clear space, and syndesmotic clear space. After satisfactory stability was obtained (stress negative mortise), incisions were irrigated and closed in layered fashion and the patient was placed in a three-sided plaster splint. All patients were instructed to remain NWB until their initial office follow-up at three weeks.

**Figure 1:**
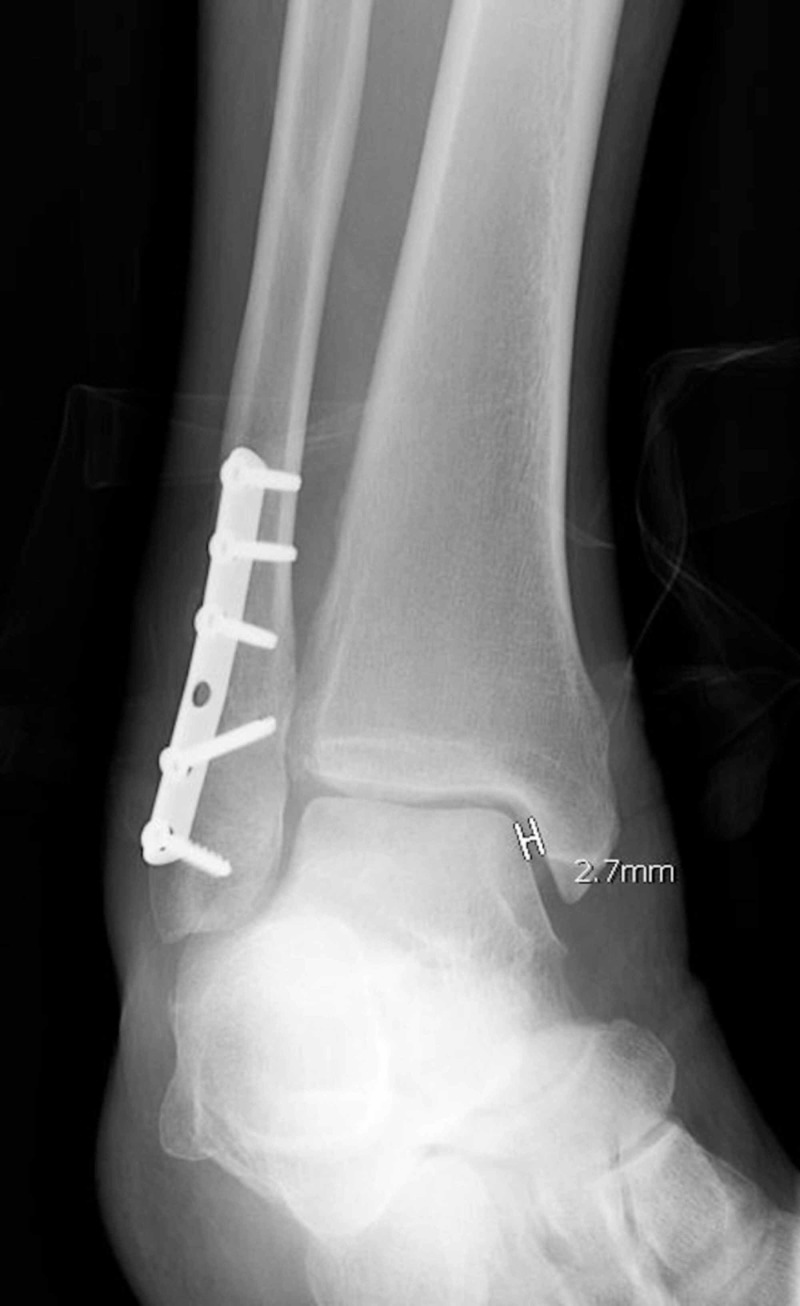
Medial clear space as measured on mortise view

Beginning at the patient’s initial three-week follow-up visit, radiographs of the ankle mortise were evaluated. These were subsequently followed at routine periods postoperatively including six weeks, twelve weeks, six months, and one year. Close attention was paid to the medial clear space, radiographic evidence of healing, and signs to suggest hardware failure (breakage). Radiographic medial clear space was measured just inferior to the medial shoulder of the talus on the ankle mortise view using the picture archiving and communication system (PACS) imaging system and was defined as normal if <5 mm (Figure 1). Radiographic healing was defined as bridging bony callus at three of four cortices or disappearance of fracture lines in fractures treated with absolute stability constructs. Loosening of hardware was also investigated and was defined as lucency about a screw or “backing out” of a screw from its formerly purchased cortex. All radiographs were reviewed by the attending orthopedic surgeon and an additional reviewer with nonunion being classified as the absence of the aforementioned signs of radiographic healing within the first six months postoperatively. While EWB was allowed strictly at three weeks postoperatively, the LWB group was allowed to weight bear after evidence of radiographic union was noted as detailed above.

Statistical analysis was performed, with means, ranges, and confidence intervals calculated for continuous variables and compared using Student’s t-tests. Frequencies were calculated for continuous variables and compared using Fisher’s exact test for increased accuracy in small proportion analysis. A significance level of p<0.05 was set as significant.

## Results

A total of 95 patients met the inclusion criteria, and of these patients, 54% were female. The EWB and LWB groups had 38 and 57 patients enrolled, respectively. Between the two groups, there were no significant differences in demographic information, including age, sex, medical comorbidities, or type of injury.

The majority of these patients (51%) suffered a fall as the primary mechanism of injury, with no differences in mechanism between the groups (p=0.22). Bimalleolar fracture pattern was seen in 66% of the patients, with the remainder being classified as bimalleolar equivalent fractures. No significant differences were found between the EWB and LWB groups with regard to any of the demographic variables listed in Table 1.

**Table 1:** Patient Demographics Absolute numbers are given as means and standard deviations, with ranges in parentheses. Categorical numbers are given as absolute numbers with percentages in parentheses.

	Delayed weight bearing (n=57)	Early weight bearing (n=38)	P value
Age (years)	46.5 ± 15.7 (18-75)	51.1 ± 13.5 (18-81)	0.18
Sex (male)	27 (47.4%)	16 (42.1%)	0.68
Injured side (left)	26 (45.6%)	14 (36.8%)	0.4
Fracture pattern			0.93
Lateral malleolus	20 (35.1%)	13 (34.2%)	
Bimalleolar	37 (64.9%)	25 (65.8%)	
Diabetes mellitus	6 (10.5%)	1 (2.6%)	0.24
Tobacco use	21 (36.8%)	10 (26.3%)	0.37
Osteoporosis medication	0	1 (2.6%)	0.4

The EWB group achieved full weight-bearing status at an average of 3.1 ± 1.4 weeks, which was significantly lower than the LWB group at 7.2 ± 2.1 weeks (p<0.01). All patients in the EWB group went to union, with time to radiographic union of 9.6 ± 3.1 weeks on average. In the LWB group, 2/57 (4%) patients were designated as nonunions after six months. The remainder of the LWB group did go on to union at a time of 8.8 ± 2.7 weeks. Union rate (p=0.51) and time to union (p=0.23) were not statistically significant between the two groups in question (Table 2).

**Table 2:** Postoperative Outcomes

Variable	Delayed weight bearing (n=57)	Early weight bearing (n=38)	P value
Time to full weight bearing (weeks)	7.23 ± 2.05	3.10 ± 1.42	<0.01
Union rate	55 (96.5%)	38 (100%)	0.51
Time to union (weeks)	8.79 ± 2.79	9.62 ± 3.13	0.23
Radiographic clear space (mm)			
Three weeks postoperatively	2.37 ± 0.87	2.14 ± 0.72	0.19
Six weeks postoperatively	2.19 ± 0.75	2.13 ± 0.70	0.7
Twelve weeks postoperatively	2.30 ± 0.94	2.14 ± 0.65	0.46
Six months postoperatively	2.27 ± 0.71	2.27 ± 0.76	0.99
One year postoperatively	2.23 ± 0.60	1.93 ± 0.81	0.33
Implant loosening			
Three weeks postoperatively	0	0	0
Six weeks postoperatively	2 (3.5%)	1 (2.6%)	0.79
Twelve weeks postoperatively	3 (5.3%)	2 (5.3%)	0.98
Six months postoperatively	3 (5.3%)	2 (5.3%)	0.98
One year postoperatively	4 (7.0%)	2 (5.3%)	0.7
Implant breakage			
Three weeks postoperatively	0	0	0
Six weeks postoperatively	2 (3.5%)	1 (2.6%)	0.79
Twelve weeks postoperatively	3 (5.3%)	4 (10.5%)	0.6
Six months postoperatively	5 (8.8%)	5 (13.1%)	0.77
One year postoperatively	5 (8.8%)	6 (15.8%)	0.5

Postoperatively, there was no significant difference in medial clear space between the EWB and LWB groups at any time interval postoperatively (p>0.1 at all time intervals). Radiographic interpretation for implant loosening was also performed at the same time intervals, and no significant differences were noted regarding implant loosening between the two groups (p>0.1 at all time intervals). Finally, implant failure was appreciated in five LWB patients and six patients in the EWB group, with no significant difference between the groups (p>0.1 at all time intervals).

## Discussion

Although a few studies have compared EWB and LWB for surgically stabilized ankle fractures and demonstrated similar functional outcomes, postoperative rehabilitation protocols have largely remained unchanged in today’s clinical practice [4,8,11,19-25]. Perhaps this relates to a lack of thorough investigation with regards to radiographic parameters other than fracture union when comparing EWB and LWB groups. In this analysis, we evaluated the medial clear space and rates of implant failure in addition to radiographic evidence of union. Other studies have corroborated that maintenance of talar position within the mortise is of critical importance in maximizing ankle function and decreasing risk for post-traumatic arthritis of the ankle joint [26-28]. Along these lines, our study suggests no increase in medial clear space at any time interval when comparing the EWB and LWB groups and no other negative sequela in the EWB cohort when evaluating fracture union, time to union, or implant failure.

Stability of the ankle mortise, as well as the ability to achieve successful osseous union, appears to be independent from the postoperative weight-bearing status following ORIF of bimalleolar ankle fractures. In this study, we found no significant difference in union rates, time to union, or implant failure in patients managed with EWB protocols compared to the more conventional LWB practices. Simanski et al. evaluated 43 patients (following bimalleolar fracture fixation) comparing EWB and LWB and demonstrated no disadvantage with EWB; it should be noted, however, that EWB was defined on average as seven weeks postoperatively [11]. This is a notably different description of EWB compared to the three weeks as described in our protocol. This further reinforces the utility of this study, as it allows the first postoperative appointment and beginning of weight bearing to coincide sooner than previously described.

Strengths of the present study include good heterogeneity between the groups. Two independent reviewers evaluated radiographs and performed medial clear space measurements in addition to making determinations in the presence of fracture union. The study follows patients past one year postoperatively providing interval-based data over that time to ensure results were consistent. As with any study, there are limitations inherent to this investigation, especially tied to its retrospective nature. Although we show that EWB does not negatively affect medial clear space, union, or hardware failure, we did not investigate clinical function or patient satisfaction. Finally, as fixation of these fractures was performed by fellowship-trained orthopedic trauma surgeons, fixation methods may differ in the community setting, although the relatively generalized principles of ankle fracture osteosynthesis should limit these differences and allow reasonable external validity. Despite the use of very similar fixation methods, an additional limitation lies within the nonrandomized structure of having one physician treat all patients that were in the EWB group, and the second who treated all patients in the LWB group. Although small, there is a possibility that differences in surgical technique could have yielded different outcomes despite varying weight-bearing protocols postoperatively.

Rehabilitation protocols and a preference for EWB versus LWB following operative stabilization of bimalleolar ankle fractures continue to vary between physicians. Traditional methodologies involving a strict NWB postoperative period until radiographic healing signs are present continue to dominate in clinical practice. This study serves to provide evidence that EWB at three weeks postoperatively does not negatively affect stability of the ankle mortise; this conclusion is validated by no change in medial clear space as well as no effect on the rate of fracture union or implant failure with EWB. Clinical consideration should be given to these findings moving forward with regard to bimalleolar and bimalleolar equivalent ankle fractures treated with ORIF. 

## Conclusions

This study found that EWB at three weeks following ORIF of bimalleolar and bimalleolar equivalent ankle fractures led to no increase in complications or nonunion rates. EWB for bimalleolar ankle fractures does not affect the radiographic medial clear space when compared to LWB. No differences in time to union, union rate, implant loosening, or failure were noted between the groups. Orthopedic surgeons should feel comfortable progressing patients’ weight-bearing status prior to six weeks postoperatively in the setting of rigid ankle ORIF without fear of implant failure or loss of reduction. Further investigations are necessary to consider the clinical impact of EWB in these fractures.
